# Association of Common Polymorphisms in the Interleukin-1 Beta Gene with Hepatocellular Carcinoma in Caucasian Patients with Chronic Hepatitis B

**DOI:** 10.3390/pathogens12010054

**Published:** 2022-12-29

**Authors:** Janett Fischer, Shuang Long, Eleni Koukoulioti, Tobias Müller, Balazs Fueloep, Renate Heyne, Mohammed Eslam, Jacob George, Fabian Finkelmeier, Oliver Waidmann, Thomas Berg, Florian van Bömmel

**Affiliations:** 1 Division of Hepatology, Department of Medicine II, Leipzig University Medical Center, 04103 Leipzig, Germany; 2 Hepatogastroenterology Unit, Second Department of Internal Medicine-Propaedeutic, Research Institute and Diabetes Center, Medical School, National and Kapodistrian University of Athens, “Attikon” University General Hospital, 12462 Athens, Greece; 3 Department of Medicine—Hepatology and Gastroenterology, Charité—Universitätsmedizin Berlin, 14197 Berlin, Germany; 4 Department of Gastroenterology, Kantonsspital Baselland, 4410 Liestal, Switzerland; 5 Liver and Study Center Checkpoint, 10969 Berlin, Germany; 6 Storr Liver Centre, Westmead Hospital and Westmead Millennium Institute for Medical Research, University Sydney, Sydney 2006, Australia; 7 Department of Gastroenterology, Hepatology and Endocrinology, University Hospital Frankfurt, 60528 Frankfurt, Germany; 8 Center for Hematology and Oncology Bethanien, 60389 Frankfurt, Germany

**Keywords:** risk variant, liver cancer, proinflammatory cytokine

## Abstract

Interleukin-1 beta (IL-1β) promotes liver disease progression and hepatocarcinogenesis in chronic hepatitis B (CHB). Single nucleotide polymorphisms (SNPs) within the promotor region of the *IL-1β* gene can affect the progression towards liver cirrhosis and hepatocellular carcinoma (HCC). **Aims:** We aimed to investigate the association of three common *IL-1β* SNPs with hepatitis B virus (HBV)-related HCC in Caucasian patients. **Method:** A Caucasian cohort of 99 patients with HBe antigen (Ag)-positive CHB, 255 patients with HBeAg-negative CHB and 278 inactive carriers (IC) were enrolled. 105 patients were diagnosed with liver cirrhosis, and 64 with HCC and cirrhosis. Genotyping of the *IL-1β* rs1143623, rs1143627 and rs16944 was performed. **Results:** The rs1143627 TT and rs16944 CC genotypes were more frequent in patients with HCC compared to patients without liver tumours (48% vs. 33%, *p* = 0.018 and 47% vs. 31%, *p* = 0.001, respectively). In multivariate analysis, the rs16944 CC genotype was independently associated with HCC (OR = 6.44 [95% CI 1.50–27.59] *p* = 0.012). The haplotype, including rs1143623 TT and rs16944 CC, was a risk factor for HCC development (OR = 1.55 [95% CI 1.04–2.32] *p* = 0.031). **Conclusions:** We identified an association of common *IL-1β* SNPs with HBV-related HCC in a Caucasian population. The effect was independent of the phases of chronic HBV infection, which are currently regarded as important HCC risk factors.

## 1. Introduction

Chronic hepatitis B virus (HBV) infection is the main risk factor for the development of liver cirrhosis and hepatocellular carcinoma (HCC). Accordingly, around 2–5% of patients with HBV-related liver cirrhosis develop HCC per year and globally, approximately 54% of HCC cases can be attributed to HBV infections [1,2]. The risk of developing HCC is variable and driven by several host factors such as the presence of liver cirrhosis, older age and male sex, as well as by HBV factors such as high HBV DNA and HBV surface antigen (HBsAg) levels, certain HBV variants and HBV genotype [3]. However, apart from those risk factors, host genomic variants have been shown to be associated with the risk of HCC development. Thus, several candidate gene-based case-control association studies have reported that common single nucleotide polymorphisms (SNPs) in various interleukin (IL) genes such as *IL-12*, *IL-10*, and *IL-4* are associated with HBV susceptibility and HBV persistence or with the risk of HBV-related HCC [4,5,6,7].

The cytokine IL-1β is known to participate in both systemic and local inflammatory processes and to mediate several immune responses [8,9]. In carcinogenesis, IL-1β is suggested to increase angiogenesis and promote tumour invasiveness and metastasis [10]. In the promoter region within the *IL-1β* gene, three SNPs were described at position rs1143623, rs1143627 and rs16944. Previous studies reported that the *IL-1β* SNPs were linked to the development and pathogenesis of numerous chronic inflammatory diseases [11,12,13] as well as the progression towards chronic hepatitis B [14,15,16].

In this study, we aimed to assess the association of the common SNPs rs1143623, rs1143627, and rs16944 in the *IL-1β* gene with the development of liver cirrhosis and HCC in a large multicenter cohort of Caucasian patients with chronic HBV infection.

## 2. Patients and Methods

### 2.1. Patients

A total of 632 patients of Caucasian origin with chronic HBV mono-infection, defined by the presence of HBsAg and HBV DNA for more than six months, were retrospectively enrolled from four academic hepatology centres in Germany (Section of Hepatology, University Hospital of Leipzig, Germany, Department of Hepatology and Gastroenterology, University Hospital Charité, Berlin, Germany and Department for Internal Medicine I/Gastroenterology and Hepatology, University Hospital Frankfurt, Frankfurt am Main, Germany) and Australia (Storr Liver Centre, Westmead Hospital and Westmead Millennium Institute for Medical Research, University Sydney), and one primary health provider (Liver and Study Centre Checkpoint, Berlin, Germany) between 2003 and 2015. All patients provided written informed consent. The study was approved by the Ethics Committees of Medical Research of the University of Leipzig and Berlin in accordance with the Declaration of Helsinki from 1975 (revision 2013) and the International Conference on Harmonization/Committee for Proprietary Medicinal Products “Good Clinical Practice” guidelines. On their first visit to the liver centres (baseline), blood samples for analysis were collected, and clinical data were assessed. The patients were monitored until 2022. Thus, liver cirrhosis or HCC development was documented during this follow-up period. If patients developed liver cirrhosis or HCC in this period, the clinical data at presentation were used for analysis.

Patients were divided into those with HBeAg-positive chronic hepatitis B (*n* = 99, HBV DNA level > 2000 IU/mL and elevated serum alanine aminotransferase [ALT] levels in the absence of secondary liver disease), those with HBeAg-negative chronic hepatitis B (*n* = 255, HBV DNA level > 2000 IU/mL and elevated serum ALT levels), and those with HBeAg-negative infection (previously termed ‘inactive carrier’ phase, IC) (*n* = 278, HBeAg-negative and HBV DNA levels < 2000 IU/mL, persistently normal serum ALT levels ULN ≤ 40 IU/L) according to the current European guidelines. [2] Caucasian ethnicity was defined as patients descended from Northern/Central or Eastern Europe (*n* = 402), the Mediterranean region (*n* = 211) or the Middle East (*n* = 19). Moreover, the patients were from academic liver centers, and selection bias cannot be excluded. The diagnosis of liver cirrhosis (LC) was based on ultrasound or computer tomography imaging to detect morphological changes such as nodular hepatic contour, an enlarged caudate lobe and left lobe lateral segment, atrophy of the right and left lobe medial segments, widening of the fissures and the porta hepatis, and regenerative nodules [17] and/or a liver biopsy (*n* = 87). Measurement of liver stiffness by transient elastography (FibroScan) for diagnosis of liver fibrosis [18] was performed in 187 patients. HCC was diagnosed by histological examination of tumor tissue or evidence on imaging. [1] The FIB-4 score can be used for the assessment of the severity of liver fibrosis and is calculated as follows: age × AST/platelet count [×10^3^/μL] × [ALT]^1/2^. The cut-off values were described previously [19]. A FIB-4 score ≤ 3.25 is representative of mild and/or moderate fibrosis, whereas a FIB-4 score > 3.25 is representative of advanced fibrosis or cirrhosis. Patients were classified into two subgroups at baseline: low scores (FIB-4 ≤ 3.25, *n* = 473) and high scores (FIB-4 > 3.25, *n* = 71).

### 2.2. Genotyping

Genomic DNA was extracted from whole blood samples with an extraction kit from QIAGEN (Hilden, Germany). Genotyping of the *IL-1β* SNPs rs1143623, rs1143627 and rs16944 was performed by real-time polymerase chain reaction (PCR) and melting curve analysis in a Light Cycler^®^ 480 System (Roche, Basel, Switzerland) using fluorescence resonance energy transfer (FRET) probes (Eurogentec, Lüttich, Belgium). Sequences of primer and probes were: rs11464323 F-5′AGGCTGCTTGGAGAGC-3′, R-5′AGTATGTGCCAGGTATCG-3′, sensor 5′TCACTCCCTTGCATAATGC-FL, anchor Atto620-GAGCGAGCACGATACCTGGC-Ph; rs114643627 F-5′GAAGCTTCCACCAATACTC-3′, R-5′TGCCTTGTGCCTCGAAG-3′, senor 5′TATGGCTTTCAAAAGCAGAAG–FL, anchor Atto620-AGGAGGCTGAGAAATTTCTCTG-Ph; and rs16944 F-5′CTTCCCACTTACAGATGG-3′, R-5′TCTGGCATTGATCTGGTTC-3′, sensor 5′CTCTGCCTCAGGAGCTC-FL and anchor Atto620-CTGTCAATTGCAGGAGCCTCTG-Ph. The PCR conditions were established as previously described [20]. Sequencing was performed with BigDye^TM^ Terminator and a capillary sequencer from Applied Biosystems (Darmstadt, Germany).

### 2.3. Determination of Cytokine Levels

Concentrations of IL-1β were measured in sera of 132 Caucasian patients with chronic HBV infection and 160 healthy Caucasian blood donors with the LEGENDplex^TM^ Human Anti-Virus Response Panel (BioLegend, Fell, Germany) according to the manufacturer’s instruction.

### 2.4. Statistical Analysis

Statistical analyses were performed using SPSS software (SPSS Inc., version 24.0, Chicago, IL, USA). The genotype distributions of the two SNPs were tested for deviations from the *Hardy*-*Weinberg Equilibrium* (HWE) [21] using the DeFinetti program with a cut-off *p*-value of 0.01.

Comparisons of the distributions of demographical characteristics between the different groups were made using the Mann–Whitney U test for continuous variables (each when adequate) and the Fishers exact test for categorical variables. Univariate and multivariate logistic regression analyses, including the factors age, sex, baseline HBV DNA level and ALT level, presence of HBeAg, IC state and presence of liver cirrhosis (when appropriate), were performed to determine the association between the SNPs and the liver disease status under dominant and recessive genetic models. Variables with *p* < 0.1 in univariate analysis were included in the multivariate logistic regression analyses.

All tests were two-sided, and *p*-values less than 0.05 were considered statistically significant. The odds ratio (OR) and the 95% confidence interval (CI) were calculated. We aimed to estimate both the recessive and additive effects of the SNPs. The structure of linkage disequilibrium (LD) was analysed with Haploview 4.2 (Broad Institute, Cambridge, MA, USA) by using the expectation–maximization (EM) algorithm. The LD is present between the single SNPs. D’ varies from 0 (complete equilibrium) to 1 (complete disequilibrium). R^2^ shows the correlation between SNPs. When R^2^ = 1, two SNPs are in perfect LD, and allelic frequencies are identical for both SNPs [21].

## 3. Results

### 3.1. Patient Characteristics and IL-1β Genotype Distribution

The baseline characteristics of the study cohort are shown in Table 1. Patients with HBeAg-negative infection (IC) included significantly fewer males compared to patients with HBeAg-positive (*p* = 1.61 × 10^−5^) and HBeAg-negative CHB (*p* = 0.0001). Furthermore, patients in the IC group developed less severe stages of liver fibrosis than patients in both CHB groups. In detail, liver cirrhosis was diagnosed in 4.0% and HCC in 4.0% of IC compared to 27.5% and 24.2% of cases with LC, and 16.1% and 12.1% cases with HCC in the HBeAg-negative and HBeAg-positive CHB groups, respectively. Alpha-fetoprotein (AFP) was measured in 115 patients (IC: *n* = 44, HBeAg-positive CHB: *n* = 10 and HBeAg-negative CHB: *n* = 61). AFP levels were significantly higher in HBeAg-negative CHB compared to IC (*p* = 0.043) but not compared to HBeAg-positive CHB (*n* = 0.705).

Additionally, one patient with HCC presented with prostate cancer, and another patient had an adenocarcinoma. In patients without HCC, the following other cancer types were diagnosed: breast cancer (*n* = 3), renal cell carcinoma (*n* = 1), bronchial cancer (*n* = 1), squamous cell carcinoma (*n* = 1) and B-cell chronic leukemia (*n* = 1).

The overall genotype distribution of *IL-1β* rs1143623 was 51% CC, 41% CG and 7% GG; rs1143627 was 35% TT, 50% CT and 15% CC; and rs16944 was 33% CC, 54% CT and 13% TT. Allele frequencies were similar to the allele frequencies in subjects of Caucasian and Asian origin analysed in the 1000 Genomes [22] and HapMap [23] projects. There were no significant differences in the genotype distribution of rs1142623, rs1143627 and rs16944 between the IC, HBeAg-negative and HBeAg-positive CHB groups.

### 3.2. Association of the IL-1β SNPs with Liver Cirrhosis

Patients with liver cirrhosis were predominantly male (90% vs. 10%, *p* = 3.85 × 10^−12^). Genotype distribution of the *IL-1β* SNPs rs1143623, rs1143627 and rs16944 were not significantly different between patients with and without ultrasound signs of liver cirrhosis in the overall cohort (Table 2). In adjusted multivariate regression analysis, the factors age (OR = 1.09 [95% CI 1.06–1.12] *p* = 1.43 × 10^−9^), male sex (OR = 5.68 [95% CI 2.49–12.98] *p* = 3.78 × 10^−5^), Fib-4 score (OR = 1.08 [95% CI 1.00–1.16] *p* = 0.040), HBeAg-positive CHB (OR = 6.56 [95% CI 1.68–25.57] *p* = 0.007), and HBeAg-negative CHB (OR = 8.56 [95% CI 2.57–28.55] *p* = 0.0005) were significantly associated with an increased likelihood of liver cirrhosis diagnosis. However, there was a significant association of the rs16944 CC genotype with the FIB-4 score > 3.25, which correctly identifies significant fibrosis with a specificity of 98–99% [19] in univariate analysis (OR = 1.72 [95% CI 1.03–2.86] *p* = 0.037). In multivariate regression analysis, adjusting for age, male sex, diabetes, and disease stage, the CC genotype did not remain independently associated with the FIB-4 score > 3.25 (OR = 1.57 [95% CI 0.85–2.89] *p* = 0.146).

### 3.3. Association of the IL-1β SNPs with HCC

Genotype distributions of the *IL-1β* rs1143627 and rs16944 were significantly different between patients with and without HCC in the overall cohort (Table 3). The TT genotype of rs1143627 and the CC genotype of rs16944 were more frequent in patients with HCC compared to non-HCC patients (rs1143627 TT: 48.4% vs. 33.1%, *p* = 0.047; rs16944 CC: 46.9% vs. 30.8%, *p* = 0.001). There were no differences in the genotype distribution of the SNP rs1146323. In univariate logistic regression analysis, an increased likelihood of HCC development was observed for the rs1143627 TT genotype (OR = 1.90 [95% CI 1.13–3.20] *p* = 0.016) and for the rs16944 CC genotype (OR = 2.39 [95% CI 1.42–4.03] *p* = 0.001) under recessive models. In adjusted multivariate regression analysis, only the rs16944 CC genotype remained independently associated with HCC (OR = 6.44 [95% CI 1.50–27.59] *p* = 0.012). Interestingly, the combination of both risk variants rs1143627 TT and rs16944 CC was present in 48% of patients with HCC compared to 28% of patients without HCC diagnosis (*p* = 7.28 × 10^−5^). This risk variant combination was associated with a higher likelihood of hepatocarcinogenesis with an OR of 2.40 (95% CI 1.42–4.04, *p* = 0.001) in univariate, and an OR of 3.47 (95% CI 1.50–8.01, *p* = 0.004) in adjusted multivariate regression analysis, respectively.

In the group of patients with liver cirrhosis (*n* = 105), the risk variants of both SNPs were more frequent in patients with HCC than those without HCC (rs1143627 TT: 41% vs. 29%, *p* = 0.016; rs16944 CC: 53% vs. 25%, *p* = 0.005). Both variants were associated with HCC in univariate analysis with an OR of 2.83 (95% CI 1.26–6.33, *p* = 0.012) and OR of 3.39 (95% CI 1.49–7.74, *p* = 0.004), respectively. In adjusted analysis, both SNP showed no independent association with HCC (Table S1). Thus, only the combination of both risk variants increased the risk of HCC development in univariate (OR = 4.63 [95% CI 1.95–10.99] *p* = 0.001) and multivariate analysis (OR = 4.73 [95% CI 1.82–12.35] *p* = 0.001).

Since HCC development occurs primarily in older patients, we performed age-stratified analyses. In the patient group with an age of ≥50 years (50-plus, *n* = 235), 80% (52/64) of all HCCs were diagnosed. Stratification for 50-plus age revealed HCC risk in patients carrying the rs16944 CC genotype with an OR of 2.44 (95% CI 1.20–4.97, *p* = 0.014) in univariate and with an OR 4.16 (95% CI 1.77–19.89, *p* = 0.001) in multivariate regression analyses (Table 4).

### 3.4. Haplotype Analysis of IL-1β rs1143627 and rs16944

Since the *IL-1β* SNPs rs1143627 and rs16944 are in moderate LD (D’ > 0.8, r^2^ < 0.8), in the overall cohort, four haplotypes exist (Table 5): rs1143627T/rs16944C (56.5%), rs1143627C/rs16944T (37.1%), rs1143627T/rs16944T (3.3%) and rs1143627C/rs16944C (3.1%). The rs1143627T/rs16944C haplotype comprising both risk variants was significantly associated with a higher risk of HCC development in the overall cohort (OR = 1.55 [95% CI 1.04–2.32] *p* = 0.031). The risk of progression towards HCC was 12% in patients carrying the TC haplotype compared to 8% of patients carrying the CT haplotype (*p* = 0.033). In the subgroup of patients with liver cirrhosis, the TC haplotype was present in 59% of patients and associated with an increased risk of HCC development with an OR of 2.03 (95% CI 1.12–3.68, *p* = 0.02).

### 3.5. Effect of rs1143627 and rs16944 on IL-1β Serum Levels

**Figure 1:**
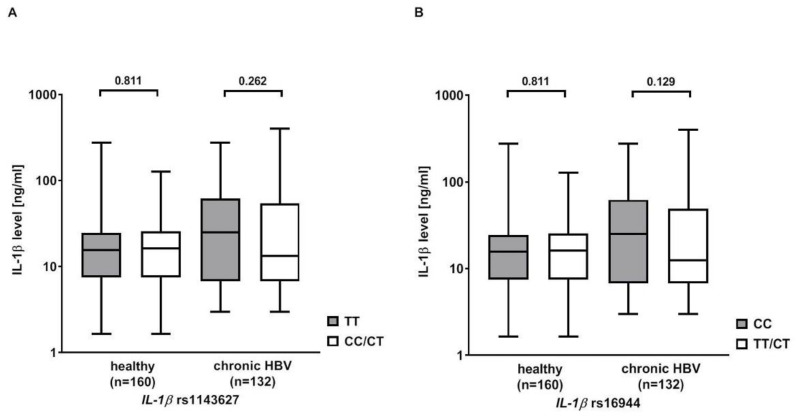
Serum levels of interleukin (IL) 1β in patients with chronic HBV infection and healthy controls (HC) stratified according to the *IL-1β* (**A**) rs1143627 and (**B**) rs16944 genotypes. Statistical analysis was performed with Mann–Whitney U Test. Columns show mean values with bars indicating standard error of mean. *Y*-axis is shown in log10.

IL-1β serum levels were measured in 132 Caucasian patients with chronic HBV infection and 160 healthy Caucasian blood donors serving as controls. The serum levels were higher in patients with chronic HBV infection than in healthy controls (40.9 ± 60.85 ng/mL vs. 22.0 ± 30.0 ng/mL, *p* = 0.081). Then, the analysis was stratified according to the rs1143627 and rs16944 genotypes. Patients with the rs1143627 TT or rs16944 CC genotype showed higher IL-1β serum levels than carriers of the rs1143627 CT/CC or rs16944 CT/TT genotypes, but significance was missed (rs1143627: 47.5 ± 63.8 ng/mL vs. 37.7 ± 59.4 ng/mL, *p* = 0.262; rs16944: 48.0 ± 62.0 ng/mL vs. 37.0 ± 60.2 ng/mL, *p* = 0.129). No differences in IL-1β serum levels were observed in the healthy controls (Figure 1).

## 4. Discussion

In the present study, we showed for the first time a strong association of the common SNPs rs1143627 and rs16944 in the *IL-1β* gene with HBV-related HCC in this large multicentre Caucasian population study. The association of the *IL-1β* polymorphisms with HCC was independent of the known risk factors, such as the presence of liver cirrhosis, older age, male sex and high HBV DNA levels [3], and independent from the phases of chronic HBV infection, in detail HBeAg-positive or HBeAg-negative CHB and HBeAg-negative infection. 

In our study, patients carrying the *IL-1β* rs16944 CC variant had more than seven times higher risk of being diagnosed with HCC than individuals carrying other variants. This finding is in accordance with previous studies performed in Asian populations in which the presence of the rs1644 CC variant increased the HCC risk up to 2.6-fold [24,25,26]—adjusting the analysis to the known risk factors of HCC development, such as the presence of liver cirrhosis, older age and male sex as well as high HBV DNA [3], a strong association with hepatocarcinogenesis was also observed in patients who were >50 years old and carried the rs16944 CC variant. Similar to the rs16944 SNP, the TT variant of *IL-1β* rs1143627 was also associated with HCC development in chronic HBV infection. However, the association was not independent of the rs16944 SNP, supporting the possible relationship between both *IL-1β* polymorphisms. The combination of both genotypes rs1143627 TT and rs16944 CC strengthened the power of HCC risk prediction. Furthermore, in haplotype analysis, the *IL-1β* rs1143627T/rs16944C haplotype, associated with increased hepatocarcinogenesis, was present in almost 70% of patients with HCC. In our population, the overall risk of HCC development was increased up to 12% in chronically infected patients carrying the *IL-1β* TC haplotype compared to 8% in patients carrying the CT haplotype. Moreover, in patients with liver cirrhosis carrying the *IL-1β* risk haplotype, the likelihood of HCC was two times higher than in carriers of the CT haplotype.

It is well known that HCC incidence varies between the different phases of chronic HBV infection. The lowest annual risk of HCC development shows patients with chronic HB infection (<1%), followed by patients with liver cirrhosis with HBeAg-negative or HBeAg-positive CHB (2–5%) [1,2,3]. In our study, we also detected the fewest number of HCCs in the IC group and no significant differences in HCC diagnosis between HBeAg-negative and HBeAg-positive CHB. The presence of liver cirrhosis was the main risk factor. However, the impact of the *IL-1β* polymorphisms was independent of the phases of chronic HBV infection, and the presence of liver cirrhosis enhanced the effect. In all subgroups, patients with HCC showed a higher prevalence of the *IL-1β* variants compared to non-HCC patients.

IL-1β regulates various immune [8,9] and inflammatory responses [27] and promotes hepatocarcinogenesis [10]. Thus, the IL-1β pathway is known to be part of the NLRP3 inflammasome, which in its active form is involved in the upregulation of fibrotic markers in the liver [28]. Indeed, significant evidence points towards IL-1β as an important mediator of the transition from liver injury to the onset of liver fibrogenesis [29]. Functional studies supported the association of the *IL1-β* TC haplotype with more severe stages of chronic liver diseases. Hence, rs1143627 SNP alters the TATA sequence in the promotor region. The T allele was associated with a five-fold higher binding activity to the transcription factors compared to the C allele [30], resulting in an elevated *IL-1β* gene expression in human monocytes [31,32], A549 cells [33] and lung tissue [34]. In an ex vivo blood stimulation assay, the *IL-1β* TC haplotype was linked to a 2–3-fold increase in the secretion levels of IL-1β after stimulation with lipopolysaccharide [35]. Furthermore, Su et al. reported that circulating IL-1β negatively affected spontaneous HBV clearance [36]. Additionally, the *IL-1β* rs1143623 C allele was demonstrated to have a higher binding to nuclear extract factors in electrophoretic mobility shift assay, suggesting a stronger promotor activity compared to the major G allele [37]. Interestingly, we only found slightly higher IL-1β serum levels in chronically infected patients carrying the rs1143627 TT or rs16944 CC genotypes compared to carriers of the other variants. However, it has been proposed that gene expression is regulated differently in blood cells and liver tissue resulting in divergent protein levels [38]. Thus, the measurement of cytokine levels in liver tissue might be more reliable.

It is still an unanswered question whether polymorphisms in the *IL-1ß* gene support HCC development via fibrogenesis alone or whether they have an intrinsic hepatocarcinogenic effect. The *IL-1ß* rs1143627 TT genotype has been shown to be associated with an increased risk for HCC in Japanese patients with hepatitis C virus infection [39]. The rs16944 SNP was also shown to be associated with a progression towards chronic hepatitis B in patients with acute HBV infection [5]. Furthermore, in Japanese patients with chronic hepatitis B, an association between *IL-1ß* polymorphisms and HBV-related histologic hepatic fibrosis could be demonstrated [40]. In contrast to Migita et al. [39], we were unable to detect any association between the *IL-1β* risk variants and ultrasound signs of liver cirrhosis. However, liver histology was available only in a small set (*n* = 87) of our retrospective population. Furthermore, the measurement of liver stiffness by FibroScan for diagnosing liver fibrosis was only performed in 178 patients [18]. Nonetheless, the FIB-4 score, which allows an approximate estimation of the presence of liver fibrosis, was associated with the *IL-1ß* rs16944 risk variant. In fact, apart from increasing the HCC risk by increased fibrogenesis, IL-1β was shown to promote hepatocarcinogenesis independently from the presence of significant fibrosis [10]. Accordingly, increased levels of IL-1β were measured in the microenvironment of tumour tissues [41] and serum samples [42]. Moreover, genetic variants in the *IL-1ß* locus were also shown to contribute to individual susceptibility in developing metabolic syndrome [43]. Metabolic factors such as obesity and diabetes and host genetic variants are reported to have a synergistic effect on HCC development in chronic hepatitis B [44].

We have studied the influence of *IL-1ß* polymorphisms in a population of HBV-infected individuals with similar HCC incidences as reported in other studies. Thus, in Caucasian individuals with chronic hepatitis B, the annual incidence of HCC is 0.3%, and 2.2% in cirrhotic patients [1,2]. The corresponding 5-year HCC cumulative incidences are 1% and 10%, respectively [45,46]. In our population, the 5-year HCC cumulative incidence was 1.3% in chronic hepatitis B and 3.8% in cirrhotic patients. We, therefore, believe that our results can be extrapolated to other populations with chronic HBV infections. However, this needs to be studied in independent cohorts.

One important limitation of our study is the lack of information on HBsAg levels, which are a major risk factor for HCC development in chronic HBV infection [3]. In our study, HBsAg levels were only available for 258 (40.8%) patients of the total cohort (*n* = 632) and only in 15 (2.3%) patients with HCC. The patients’ data were retrospectively obtained from medical records dating back to 1996 when the quantitative determination of HBsAg levels had not yet been implemented in the daily practice and had not been routinely conducted. Thus, in upcoming studies focusing on the association of host genetics with hepatocarcinogenesis in CHB, HBsAg levels have to be included in the analysis, especially since it has been shown that HBsAg levels correlate with the phases of HBV infection [47]. Furthermore, alpha-fetoprotein measurement, a tumour marker supporting diagnosis of liver cancer, was performed in only 115 (18.2%) patients.

## 5. Conclusions

In conclusion, our study shows for the first time that the rs1143627T/rs16944C haplotype of the *IL-1β* gene is associated with HCC development in chronic HBV infection in a Caucasian population. Therefore, the polymorphic positions of the *IL-1β* gene may be important factors promoting hepatocarcinogenesis in several populations with different genetic backgrounds. Whether the increase of the HCC risk is mediated by an increase in inflammation or fibrogenesis or by a direct cancerogenic effect needs to be further shown in functional analyses. Determination of the *IL-1β* SNPs in individualized medicine might improve stratification algorithms and might increase the identification of patients with an increased risk of HCC development. Nevertheless, further large population-based studies in HBV-related HCC, as well as in other liver cancers and functional analyses, are needed to enlighten the impact of genetic variations in hepatocarcinogenesis.

## Figures and Tables

**Table 1:** Baseline characteristics of the patients with HBeAg-positive or HBeAg-negative chronic hepatitis B (CHB) or HBeAg-negative infection (IC).

Baseline Parameter	HBeAg-Negative CHB (*n* = 255)	HBeAg-Positive CHB (*n* = 99)	IC State (*n* = 278)	HBeAg-Negative vs. HBeAg-Positive CHB ‡	HBeAg-Negative CHB vs. IC State ‡	HBeAg-Positive CHB vs. IC State ‡
Age (years) †	56.7 ± 13.5	53.9 ± 14.3	51.4 ± 13.5	0.093	2.18 × 10^−5^	0.188
Male	174 (68.2%)	76 (76.8%)	144 (51.8%)	0.121	0.0001	1.61 × 10^−5^
HBV DNA (log10 IU/mL) †	3.6 ± 2.5	6.1 ± 2.2	2.0 ± 1.2	1.82 × 10^−16^	6.54 × 10^−16^	3.06 × 10^−34^
ALT (IU/mL) †	87.5 ± 204.2	79.2 ± 104.1	31.6 ± 25.0	0.044	1.52 × 10^−15^	3.67 × 10^−14^
Diabetes	23 (9.0%)	9 (9.1%)	22 (7.9%)	1.000	0.755	0.676
FIB-4 score †	2.98 ± 6.41	1.99 ± 2.86	1.18 ± 0.80	0.054	4.20 × 10^−5^	0.329
Liver cirrhosis	70 (27.5%)	24 (24.2%)	11 (4.0%)	0.566	0.003	0.003
HCC	41 (16.1%)	12 (12.1%)	11 (4.0%)	0.733	0.001	0.031
AFP (*n* = 115)	286.4 ± 1421.4	4.8 ± 4.7	4.5 ± 8.2	0.705	0.043	0.542
NUC treatment	214 (83.9%)	94 (95.0%)	52 (18.7%)	0.005	5.57 × 10^−55^	8.22 × 10^−44^
Descent Northern/Central or Eastern Europe	174 (68.2%)	65 (65.7%)	163 (58.6%)			
Middle East	4 (1.6%)	2 (2.0%)	13 (4.7%)	0.776	0.020	0.365
Mediterranean	77 (30.2%)	32 (32.3%)	102 (36.7%)			

† mean ± standard deviation, ‡ *p*-value, AFP: alpha-fetoprotein, Ag: antigen, ALT: alanine aminotransferase, CHB: chronic hepatitis B, FIB-4: fibrosis-4, HCC: hepatocellular carcinoma, IC: HBeAg-negative infection, NUC: nucleoside/nucleotide, IU: international units. Comparisons of continuous variables were made using the Mann–Whitney U test. Categorical variables were compared with the Fishers exact test.

**Table 2:** Genotype distribution of the *IL-1β* SNPs in patients with and without liver cirrhosis and the association with liver cirrhosis using logistic regression analysis.

IL1-β		LC (*n* = 105)	No LC (*n* = 527)	Unadjusted OR [95% CI]	*p*-Value	Adjusted OR [95% CI]	*p*-Value
rs1143623	CC	56 (53.3%)	268 (50.9%)	REF			
	CG	40 (38.1%)	221 (41.9%)	0.87 [0.56–1.35]	0.252		
	GG	9 (8.6%)	38 (7.2%)	1.13 [0.52–2.48]	0.753		
	GG/CG vs. CC			0.91 [0.60–1.38]	0.643		
	GG vs. CG/CC			1.21 [0.57–2.57]	0.628		
rs1143627	CC	16 (15.2%)	81 (15.4%)	REF			
	CT	47 (44.8%)	269 (51.0%)	0.89 [0.48–1.64]	0.570		
	TT	42 (40.0%)	177 (33.6%)	1.20 [0.64–2.26]	0.698		
	TT/CT vs. CC			1.01 [0.56–1.81]	0.973		
	TT vs. CT/CC			1.32 [0.86–2.03]	0.208		
rs16944	TT	14 (13.4%)	71 (13.5%)	REF			
	CT	51 (54.0%)	290 (55.0%)	0.88 [0.46–1.68]	0.996		
	CC	40 (32.6%)	166 (31.5%)	1.19 [0.61–2.33]	0.610		
	CC/CT vs. TT			1.00 [0.54–1.84]	0.989		
	CC vs. CT/TT			1.32 [0.85–2.03]	0.217		
Male sex		95 (90.5%)	299 (56.7%)	**7.24 [3.69–14.21]**	**8.50 × 10^−9^**	**5.68 [2.49–12.98]**	**3.78 × 10^−5^**
Age (years) †		65.6 ± 10.2	51.6 ± 13.2	**1.10 [1.07–1.12]**	**3.29 × 10^−17^**	**1.09 [1.06–1.12]**	**1.43 × 10^−9^**
HBV DNA (log10 IU/mL) †		3.59 ± 2.95	3.33 ± 2.26	1.05 [0.95–1.15]	0.339		
ALT (IU/mL) †		119.8 ± 289.8	50.0 ± 77.4	**1.00 [1.00–1.01]**	**0.0004**	1.00 [1.00–1.00]	0.377
Diabetes		19 (18.1%)	35 (6.6%)	**3.11 [1.70–5.68]**	**0.0002**	1.90 [0.83–4.34]	0.127
FIB-4 score		5.20 ± 5.17	1.46 ± 4.03	**1.37 [1.23–1.52]**	**1.17 × 10^−8^**	**1.08 [1.00–1.16]**	**0.040**
IC		11 (10.4%)	267 (50.7%)	REF			
HBeAg-positive CHB		24 (22.9%)	75 (14.2%)	**7.77 [3.64–16.58]**	**1.17 × 10^−7^**	**6.56 [1.68–25.57]**	**0.007**
HBeAg-negative CHB		70 (66.7%)	185 (35.1%)	**9.18 [4.73–17.82]**	**8.46 × 10^−11^**	**8.56 [2.57–28.55]**	**0.0005**
NUC treatment		82 (78.1%)	278 (52.8%)	**3.19 [1.95–5.23]**	**3.93 × 10^−6^**	0.99 [0.31–3.11]	0.980
Descent Northern/Central or Eastern Europe		90 (85.7%)	312 (59.2%)	**4.39 [2.39–8.07]**	**1.84 × 10^−6^**	1.95 [0.92–4.14]	0.083
Middle East		2 (1.9%)	17 (3.2%)	1.79 [0.37–8.60]	0.466	1.44 [0.15–14.37]	0.755
Mediterranean		13 (12.4%)	198 (37.6%)	REF			

† mean ± standard deviation, Ag: antigen, ALT: alanine aminotransferase, CHB: chronic hepatitis B, CI: confidence interval, FIB-4: fibrosis-4, IC: HBeAg-negative infection, IU: international units, LC: liver cirrhosis, NUC: nucleoside/nucleotide, OR: odds ratio, REF: reference.

**Table 3:** Genotype distribution of the *IL-1β* SNPs in patients with and without HCC and the association with HCC using logistic regression analysis.

IL1-β		HCC (n = 64)	No HCC (n = 568)	Unadjusted OR [95% CI]	*p*-Value	Adjusted OR [95% CI]	*p*-Value
rs1143623	CC	34 (53.1%)	290 (51.1%)	REF			
	CG	26 (40.6%)	235 (41.4%)	0.94 [0.55–1.62]	0.833		
	GG	4 (6.3%)	43 (7.6%)	0.79 [0.27–2.35]	0.676		
	GG/CG vs. CC			0.92 [0.55–1.55]	0.754		
	GG vs. CG/CC			0.81 [0.49–2.02]	0.703		
rs1143627	CC	9 (14.1%)	88 (15.5%)	REF		REF	
	CT	24 (37.5%)	292 (51.4%)	0.81 [0.36–1.79]	0.593		
	TT	31 (48.4%)	188 (33.1%)	1.61 [0.74–3.53]	0.232		
	TT/CT vs. CC			1.12 [0.53–2.35]	0.978		
	TT vs. CT/CC			**1.90 [1.13–3.20]**	**0.016**	0.52 [0.12–2.19]	0.370
rs16944	TT	7 (10.9%)	77 (13.6%)	REF		REF	
	CT	27 (42.2%)	316 (55.6%)	0.84 [0.35–2.01]	0.833		
	CC	30 (46.9%)	175 (30.8%)	2.07 [0.88–4.90]	0.096		
	CC/CT vs. TT			1.28 [0.56–2.90]	0.559		
	CC vs. CT/TT			**2.39 [1.42–4.03]**	**0.001**	**6.44 [1.50–27.59]**	**0.012**
Male sex		58 (90.6%)	336 (59.2%)	**6.68 [2.83–15.73]**	**1.42 × 10^−5^**	**5.86 [1.47–23.31]**	**0.012**
Age (years) †		64.4 ± 11.2	52.7 ± 13.5	**1.07 [1.05–1.09]**	**7.72 × 10^−9^**	**1.06 [1.02–1.10]**	**0.005**
HBV DNA (log10 IU/mL) †		3.57 ± 2.61	3.35 ± 2.36	1.04 [0.92–1.17]	0.532		
ALT (IU/mL) †		94.0 ± 141.4	58.8 ± 140.8	1.00 [1.00–1.00]	0.188		
Diabetes		9 (14.1%)	45 (7.9%)	1.90 [0.88–4.10]	0.101		
IC		11 (17.2%)	267 (47.0%)	REF			
HBeAg-positive CHB		12 (18.7%)	87 (15.3%)	**3.34 [1.43–7.86]**	**0.006**	1.65 [0.37–7.29]	0.508
HBeAg-negative CHB		41 (63.1%)	214 (37.7%)	**4.65 [2.33–9.27]**	**1.25 × 10^−5^**	2.50 [0.72–8.73]	0.150
FIB-4 score		4.11 ± 3.86	1.90 ± 4.47	**1.06 [1.01–1.12]**	**0.029**	0.93 [0.84–1.04]	0.187
Presence of LC		49 (76.6%)	56 (9.9%)	**29.87 [15.74–56.69]**	**2.76 × 10^−25^**	**17.20 [6.27–47.19]**	**3.31 × 10^−8^**
NUC treatment		40 (62.5%)	320 (56.3%)	1.29 [0.76–2.20]	0.346		
Descent Northern/Central or Eastern Europe		59 (92.2%)	343 (60.4%)	**8.90 [3.19–24.87]**	**3.30 × 10^−5^**	2.63 [0.76–9.12]	0.127
Middle East		1 (1.6%)	18 (3.2%)	2.88 [0.31–27.10]	0.356	3.20 [0.23–44.33]	0.386
Mediterranean		4 (6.3%)	207 (36.4%)				

† mean ± standard deviation, ALT: alanine aminotransferase, Ag: antigen, CHB: chronic hepatitis B, CI: confidence interval, FIB-4: fibrosis-4, HCC: hepatocellular carcinoma, IC: HBeAg-negative infection, IU: international units, LC: liver cirrhosis, NUC: nucleoside/nucleotide, OR: odds ratio, REF: reference.

**Table 4:** Genotype distribution of the *IL-1β* SNPs in 50-plus patients (*n* = 235) with and without HCC and the association with HCC using logistic regression analysis.

IL1-β		HCC (n = 52)	No HCC (n = 183)	Unadjusted OR [95% CI]	*p*-Value	Adjusted OR [95% CI]	*p*-Value
rs1143623	CC	28 (53.8%)	97 (53.0%)	REF			
	CG	21 (40.4%)	71 (38.8%)	1.03 [0.54–1.95]	0.941		
	GG	3 (5.8%)	15 (8.2%)	0.69 [0.19–2.57]	0.583		
	GG/CG vs. CC			0.97 [0.52–1.79]	0.915		
	GG vs. CG/CC			0.69 [0.19–2.47]	0.563		
rs1143627	CC	6 (11.5%)	23 (12.6%)	REF			
	CT	21 (40.4%)	92 (50.3%)	0.88 [0.32–2.42]	0.797		
	TT	25 (48.1%)	68 (37.2%)	1.41 [0.52–3.86]	0.505		
	TT/CT vs. CC			1.40 [0.42–2.87]	0.842		
	TT vs. CT/CC			1.57 [0.84–2.91]	0.157		
rs16944	TT	4 (7.7%)	21 (11.5%)	REF		REF	
	CT	21 (40.4%)	99 (54.1%)	1.11 [0.35–3.58]	0.857		
	CC	27 (51.9%)	63 (34.4%)	2.25 [0.71–7.18]	0.171		
	CC/CT vs. TT			1.95 [0.51–4.75]	0.438		
	CC vs. CT/TT			**2.06 [1.10–3.84]**	**0.023**	**4.16 [1.79–9.71]**	**0.001**
Male sex		41 (91.1%)	106 (57.9%)	**8.72 [3.02–25.20]**	**6.38 × 10^−5^**	**5.94 [1.77–19.89]**	**0.004**
Age (years) †		68.3 ± 8.1	68.3 ± 6.7	1.00 [0.95–1.04]	0.899		
HBV DNA (log10 IU/mL) †		3.55 ± 2.50	3.30 ± 2.62	1.01 [0.89–1.15]	0.856		
ALT (IU/mL) †		94.0 ± 141.4	58.8 ± 140.8	1.00 [1.00–1.00]	0.206		
Diabetes		8 (15.4%)	31 (16.9%)	0.52 [0.19–1.42]	0.202		
IC state		10 (19.2%)	74 (40.4%)	REF			
HBeAg-positive CHB		9 (17.3%)	26 (14.2%)	2.56 [0.94–7.00]	0.067	0.74 [0.20–2.72]	0.652
HBeAg-negative CHB		33 (63.5%)	83 (45.4%)	**2.94 [1.36–6.38]**	**0.006**	0.68 [0.24–1.94]	0.474
FIB-4 score		4.12 ± 2.43	3.30 ± 6.97	1.02 [0.97–1.06]	0.487		
Presence of LC		42 (80.8%)	37 (20.2%)	**16.57 [7.61–36.09]**	**1.54 × 10^−12^**	**16.90 [6.49–44.00]**	**6.99 × 10^−9^**
NUC treatment		33 (63.5%)	118 (64.5%)	0.96 [0.50–1.82]	0.892		
Descent Northern/Central or Eastern Europe		47 (90.4%)	151 (82.5%)	2.33 [0.78–6.97]	0.129		
Middle East		7 (7.7%)	30 (16.4%)	3.75 [0.27–51.37]	0.322		
Mediterranean		1 (1.9%)	2 (1.1%)	REF			

† mean ± standard deviation, ALT: alanine aminotransferase, Ag: antigen, CHB: chronic hepatitis B, CI: confidence interval, FIB-4: fibrosis-4, HCC: hepatocellular carcinoma, IC: HBeAg-negative infection, IU: international units, LC: liver cirrhosis, NUC: nucleoside/nucleotide, OR: odds ratio, REF: reference.

**Table 5:** Haplotype frequencies of *IL-1β* rs1143627/rs16944 and association with HCC in the study cohort using logistic regression analysis.

rs1143627/rs16944 Haplotypes	Frequency			OR [95% CI]	*p*-Value
	Overall	No HCC	HCC		
TC	0.565	0.553	0.672	**1.55 [1.04–2.32]**	**0.031**
CT	0.371	0.379	0.297	REF	REF
CC	0.033	0.035	0.031	1.13 [0.39–3.34]	0.819
TT	0.031	0.033	0	0	0.998

CI: confidence interval, HCC: hepatocellular carcinoma, OR: odds ratio, REF: reference.

## Data Availability

The data presented in this study are available on request from the corresponding author.

## Supplementary Materials

The following supporting information can be downloaded at: https://www.mdpi.com/article/10.3390/pathogens12010054/s1, Table S1: Genotype distribution of the *IL-1β* SNPs in patients with liver cirrhosis (*n* = 105) with and without HCC and the association with HCC using logistic regression analysis.

## Abbreviations

AFP: alpha-fetoprotein, ALT: alanine aminotransferase, CHB: chronic hepatitis B, CI confidence interval, DNA: deoxyribonucleic acid, EM: expectation–maximization: FIB-4 score: fibrosis-4 score, FL: fluorescein, FRET: fluorescence resonance energy transfer, HBeAg: HBV envelope antigen, HBsAg: HBV surface antigen, HBV: hepatitis B virus, HCC: hepatocellular carcinoma, HCV: hepatitis C virus, HDV: hepatitis delta virus, HIV: human immunodeficiency virus, HWE: Hardy–Weinberg Equilibrium, IC: inactive carrier, IL: interleukin, IU: international units, LD: linkage disequilibrium, LC: liver cirrhosis, N: number, NLR3P: NLR family pyrin domain containing 3, OR: odds ratio, PCR: real-time polymerase chain reaction, Ph: phosphate, REF: reference, SNP: single nucleotide polymorphism.
