# Supplementary material for: Association of Common Polymorphisms in the Interleukin-1 Beta Gene with Hepatocellular Carcinoma in Caucasian Patients with Chronic Hepatitis B
Source: Pathogens. 2022 Dec 29;12(1):54. doi: 10.3390/pathogens12010054 (PMC9861021; doi:10.3390/pathogens12010054)
Supplement: Supplementary file 1 [file pathogens-12-00054-s001.zip › pathogens-2050246-supplementary.pdf]

Supplementary Material

Association of Common Polymorphisms in the Interleukin-1 Beta Gene with Hepatocellular Carcinoma in Caucasian Patients with Chronic Hepatitis B

Janett Fischer, Shuang Long, Eleni Koukoulioti, Tobias Müller, Balazs Fueloep, Renate Heyne, Mohammed Eslam, Jacob George, Fabian Finkelmeier, Oliver Waidmann, Thomas Berg, Florian van Bömmel

Table of contents

Supplementary Results

Table S1 2

Table S1: Genotype distribution of the *IL-1β* SNPs in patients with liver cirrhosis (n=105) with and without HCC and the association with HCC using logistic regression analysis

IL1-β		HCC (n=49)	No HCC (n=56)	Unadjusted OR [95% CI]	P-value	Adjusted OR [95% CI]	P-value
rs1143623	CC	29 (59.2%)	27 (49.2%)	REF			
	CG	18 (36.7%)	22 (39.3%)	0.76 [0.34-1.73]	0.512		
	GG	2 (4.1%)	7 (12.5%)	0.27 [0.05-1.39]	0.117		
	GG/CG vs. CC			0.64 [0.30-1.39]	0.262		
	GG vs. CG/CC			0.30 [0.06-1.51]	0.143		
rs1143627	CC	5 (10.2%)	11 (19.6%)	REF		REF	
	CT	18 (36.7%)	29 (51.8%)	1.32 [0.41-4.58]	0.614		
	TT	26 (53.1%)	16 (28.6%)	3.58 [1.05-12.19]	0.042		
	TT/CT vs. CC			2.15 [0.69-6.70]	0.186		
	TT vs. CT/CC			**2.83 [1.26-6.33]**	**0.012**	1.40 [0.28-6.98]	0.685
rs16944	TT	5 (10.2%)	9 (16.1%)	REF		REF	
	CT	18 (36.7%)	33 (58.9%)	0.98 [0.29-3.38]	0.977		
	CC	26 (53.1%)	14 (25.0%)	3.34 [0.94-1.92]	0.171		
	CC/CT vs. TT			1.69 [0.52-5.42]	0.438		
	CC vs. CT/TT			**3.39 [1.49-7.74]**	**0.004**	2.66 [0.53-13.46]	0.238
Male sex		46 (93.9%)	46 (82.2%)	2.19 [0.53-8.98]	0.276		
Age (years)†		66.0±9.9	65.4±10.5	1.01 [0.97-1.04]	0.781		
HBV DNA (log10 IU/ml)†		3.36±2.65	3.75±3.16	0.96 [0.83-1.10]	0.539		
ALT (IU/ml)†		97.2±139.7	140.0±376.9	1.00 [1.00-1.00]	0.473		
Diabetes		8 (16.3%)	11 (19.6%)	0.57 [0.19-1.68]	0.308		
IC state		7 (14.3%)	4 (7.1%)	REF			
HBeAg-positive CHB		6 (12.2%)	26 (46.4%)	0.19 [0.04-0.87]	0.034	0.84 [0.10-7.35]	0.877
HBeAg-negative CHB		36 (73.5%)	34 (60.7%)	0.61 [0.16-2.25]	0.454	3.10 [0.41-23.68]	0.276
FIB-4 score		4.52±4.09	5.69±5.80	0.95 [0.87-1.04]	0.294		
NUC treatment		31 (63.3%)	51 (91.1%)	**0.17 [0.06-0.50]**	**0.001**	**0.10 [0.02-0.49]**	**0.005**
Northern/Central or Eastern Europe		45 (91.9%)	(80.3%)	3.33 [0.87-12.92]	0.082	1.96 [90.45-8.60]	0.374

Middle East	3 (6.1%)	10 (17.9%)	3.33 [0.16-70.91]	0.440	3.86 [0.10-154.79]	0.474
Mediterranean region	1 (2.0%)	1 (1.8%)	REF			

† mean ± standard deviation, ALT: alanine aminotransferase, Ag: antigen, CHB: chronic hepatitis B, CI: confidence interval, FIB-4: fibrosis-4, HCC: hepatocellular carcinoma, IC: HBeAg-negative infection, IU: international units, LC: liver cirrhosis, NUC: nucleoside/nucleotide, OR: odds ratio, REF: reference
